# Efficacy and Safety of Triple Combination Cystic Fibrosis Transmembrane Conductance Regulator Modulators in Patients With Cystic Fibrosis: A Meta-Analysis of Randomized Controlled Trials

**DOI:** 10.3389/fphar.2022.863280

**Published:** 2022-03-14

**Authors:** Yizi Wang, Bin Ma, Wenya Li, Peiwen Li

**Affiliations:** ^1^ Department of Obstetrics and Gynecology, Shengjing Hospital of China Medical University, Shenyang, China; ^2^ Department of Colorectal Surgery, Liaoning Cancer Hospital and Institute, Cancer Hospital of China Medical University, Shenyang, China; ^3^ Department of Thoracic Surgery, The First Hospital of China Medical University, Shenyang, China

**Keywords:** cystic fibrosis transmembrane conductance regulator, cystic fibrosis, CFTR, corrector, potentiator

## Abstract

**Background:** Cystic fibrosis is a rare, recessive, progressive genetic disease caused by dysfunction of the cystic fibrosis transmembrane conductance regulator (CFTR) protein. Small molecules have recently been developed to treat the molecular consequences of CFTR mutations and restore CFTR protein function. However, the data on triple combination therapy (mainly from Vertex Pharmaceuticals, which is most tested in clinical trials) are limited. This meta-analysis was aimed to assess the efficacy and safety of this therapy according to different mutation genotypes and comparators.

**Methods:** Relevant publications were identified through searching several medical databases before 31 December 2021. The primary outcomes of ppFEV_1_, sweat chloride concentration and Cystic Fibrosis Questionnaire-Revised (CFQ-R) score were pooled and analyzed. The secondary outcomes were adverse events in triple combination therapy.

**Results:** Six randomized controlled trials were eligible for analysis. The total outcome of the ppFEV1 change was higher with triple combination therapy than triple placebo or active control (mean difference, MD, 13.6% and 8.74%, respectively). The pooled result of sweat chloride concentrations with triple combination therapy was lower than that of triple placebo or active control (MD, −44.13 and −39.26, respectively). The pooled estimate of the CFQ-R score was higher with triple combination therapy than triple placebo or active control (MD, 19.8% and 14.63%, respectively). No clear differences in adverse events were found between triple combination therapy and the control (placebo or active control).

**Conclusion:** CFTR modulators in triple combination achieve better clinical results than placebo and active control, and result in comparable adverse events.

**Systematic Review Registration:** https://www.crd.york.ac.uk/prospero/display_record.php?ID=CRD42021293402, identifier PROSPERO 2021 CRD42021293402.

## Introduction

Cystic fibrosis (CF) is a rare autosomal recessive, progressive genetic disease caused by dysfunction of the CF transmembrane conductance regulator (CFTR) protein. CFTR is responsible for transporting anions, such as chloride and bicarbonate, and is located at the apical surfaces of epithelial cells. If the quantity and/or function of CFTR is diminished, loss of chloride secretion and deficient fluid transport result (Habib et al., 2019), thus ultimately inducing abnormal mucus secretion and multiorgan dysfunction, including pancreatic insufficiency and airway infection and obstruction (Elborn, 2016). The chronic airway impairment leads to progressive lung damage and respiratory failure, and eventually premature death (Heijerman et al., 2019).

Bialleic mutations in CFTR genes cause CF, and more than 2000 genetic variants have been found. The most common mutation is the p.Phe508del CFTR mutation, which is found in 90% of caucasian population (Habib et al., 2019). The p.Phe508del CFTR mutation causes severe dysfunction in CFTR processing and trafficking, thus limiting the quantity and function of CFTR at the cell surface (Dalemans et al., 1991). Nearly 50% of patients have homozygous p.Phe508del CFTR mutations (p.Phe508del-p.Phe508del genotype, F/F), and almost 33% have heterozygous minimal-function CFTR mutations (p.Phe508del minimal-function, F/MF). Another category of CFTR mutations resulting in lesser impairment of CFTR protein activity is residual function mutations (RF), including some genetic mutations associated with the CFTR protein channel-gating defects, denoted gating mutations (Barry et al., 2021). Most patients with these residual function (F/RF) or gating (F-gating) CFTR mutations are heterozygous for the p.Phe508del mutation (Barry et al., 2021).

Recently, small molecules have been developed to treat the molecular consequences of CFTR mutations and restore CFTR protein function (Davies et al., 2018; Keating et al., 2018; Heijerman et al., 2019; Middleton et al., 2019; Barry et al., 2021). Generally, the modulators can be classified as CFTR potentiators (e.g., ivacaftor), which augment the gating of mutant CFTR protein, or first-generation CFTR correctors (e.g., lumacaftor and tezacaftor), which aid in processing and trafficking of the protein to the cell surface (Heijerman et al., 2019; Barry et al., 2021). A single modulator regimen (CFTR potentiator) (Ramsey et al., 2011; De Boeck et al., 2014) or a combination of two modulator regimens (Boyle et al., 2014; Rowe et al., 2017) (CFTR potentiator and CFTR corrector) has been found to ameliorate sweat chloride, lung function, respiratory-related quality of life, bodyweight, and pulmonary exacerbation. However, neither of these treatments fully restores function to the p.Phe508del CFTR protein. Therefore, more effective CFTR modulations are needed to treat the underlying cause of CF (Davies et al., 2018).

Recently, a next-generation corrector [VX-659 or elexacaftor (previously known as VX-445)] with a different structure and mechanism of action, has been found to increase CFTR processing, trafficking and function *in vitro* (Veit et al., 2020; Becq et al., 2022). The combination of a next-generation corrector and tezacaftor increases the efficacy of CFTR function to a greater extent than either compound alone (Davies et al., 2018); moreover, ivacaftor further potentiates chloride transport. However, the data on triple combination therapy (next-generation corrector plus corrector plus potentiator) are limited. This meta-analysis examines current studies on triple combination therapy and assesses the available data in terms of efficacy and safety, according to different mutation genotypes and comparators.

## Methods

### Study Search

This meta-analysis was performed according to the Preferred Reporting Items for Systematic Reviews and Meta-analyses (PRISMA) guidelines (Vrabel, 2009). The checklist is presented in Supplementary Table S1. The literature search was performed through PubMed, Web of Science and Cochrane Library on 31 Dec 2021. The search terms and queries are presented in Supplementary Table S2. This meta-analysis was registered at PROSPERO (CRD42021293402).

### Study Selection and Eligibility Criteria

Relevant studies were collected, and duplicates were removed (identification). According to the titles and abstracts, we selected the studies relevant to our analysis for full-text review (screening). Studies were screened according to the inclusion and exclusion criteria. If multiple studies reported the same outcomes based on the same patient population or cases with any overlapping information, we included only the most informative study. An additional search was performed on the references of the included studies to further identify potentially eligible studies.

The inclusion criteria were as follows: 1) population: patients diagnosed with CF with at least one p.Phe508del CFTR mutation; 2) intervention: patients who underwent triple combination therapy (next-generation corrector plus corrector plus potentiator) for CF; 3) comparison: patients who underwent placebo treatment or active-control therapy; 4) outcomes: primary outcomes included the absolute change from baseline in predicted forced expiratory volume in 1 s (ppFEV_1_), absolute change from baseline in sweat chloride concentration and absolute change from baseline in Cystic Fibrosis Questionnaire-Revised (CFQ-R) respiratory domain score; secondary outcomes included adverse events; and 5) study design: randomized controlled trials (RCTs).

The exclusion criteria were as follows: 1) case reports or reviews; 2) single arm studies; 3) no reporting of outcomes of interest; 4) studies published in languages other than English; 5) preclinical studies or experiments *in vitro*.

### Data Collection

A formalized table was independently used by Y.Z.W. and P.W.L to extract data from each paper. The following information was included: 1) authors; 2) publication year; 3) study design; 4) setting (single center/multicenter); 5) enrollment period; 6) number of patients; 7) components of triple combination therapy; 8) components of active control therapy; 9) absolute change in ppFEV_1_ (if dose differed, only data from the highest dose was collected); 10) absolute change in sweat chloride concentration (if dose differed, only data from the highest dose was collected); 11) absolute change in CFQ-R score (if dose differed, only data from the highest dose was collected); 12) any adverse events; and 13) p.Phe508del mutation type.

### Assessment of the Risk of Bias in the Included Studies

Cochrane analysis was conducted to assess the risk of bias in the RCTs (Higgins and Green, 2013). Five aspects of bias (selection bias, performance bias, detection bias, attrition bias and reporting bias) were evaluated.

### Statistical Analysis

RevMan 5.3 (Cochrane) was used for statistical analysis. The Mantel-Haenszel random effects model and risk ratio (RR) were used for binary results, and the inverse variance method was used for continuous outcomes (Higgins et al., 2003). I^2^ was used to evaluate heterogeneity, and I^2^ > 50% and *p* < 0.05 were considered thresholds for significant heterogeneity. All statistical values are reported with 95% confidence intervals (CIs). Subgroup analysis was conducted if the heterogeneity was significant.

## Results

### Search Results

**FIGURE 1:**
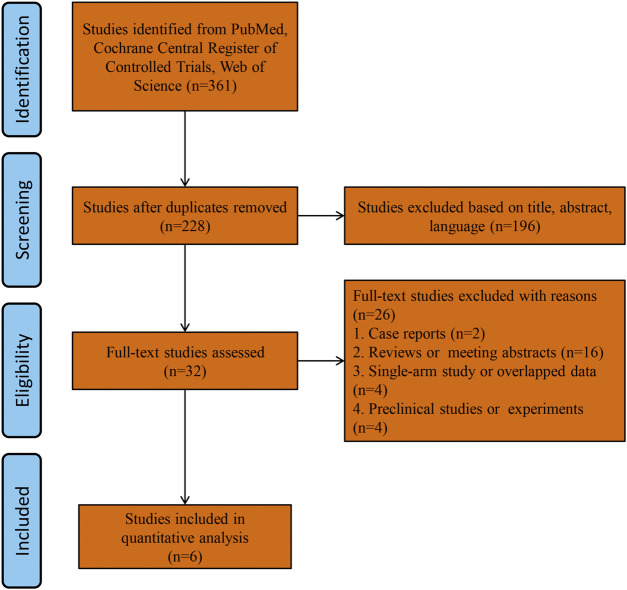
Flow chart of this meta-analysis.

A total of 361 studies were found by searching the PubMed, Cochrane Library and Web of Science databases. The study flowchart is shown in Figure 1. A total of 133 duplicate studies were excluded, and an additional 196 studies were removed for reasons associated with the title, abstract and language. Thirty-two records were eligible for full text review. Two cases series or reports studies were excluded. Sixteen studies were excluded for being reviews or meeting abstracts. Four studies were excluded for overlapping patients or being single arm studies, and four studies were excluded for being preclinical studies or experiments. Finally, six RCTs were included in the final analysis (Davies et al., 2018; Keating et al., 2018: Heijerman et al., 2019; Middleton et al., 2019; Barry et al., 2021; Sutharsan et al., 2021), all of which were multicenter RCTs. The main characteristics of the included studies are shown in Table 1. Five included studies used the same triple combination therapy (Keating et al., 2018; Heijerman et al., 2019; Middleton et al., 2019; Barry et al., 2021; Sutharsan et al., 2021) (elexacaftor-tezacaftor-ivacaftor, ELX-TEZ-IVA), and one study (Davies et al., 2018) used VX659-TEZ-IVA as the triple combination therapy. Two studies (Davies et al., 2018; Keating et al., 2018) used triple placebo or active-control as the comparator, three studies used only active control as the comparator (Heijerman et al., 2019; Barry et al., 2021; Sutharsan et al., 2021), and one study used only triple placebo as the comparator (Middleton et al., 2019).

**TABLE 1:** The main characteristics of included studies.

Author	Year	Setting	Treatment duration	Triple therapy	Placebo/Acitive placebo	No. of patients included in analysis		Genotypes
						Triple therapy	Placebo or active placebo	
Davies	2018	Multicenter	4 weeks	VX-659(400 mg)	Triple placebo or Placebo + TEZ(100 mg)+IVA(300 mg)	40	28	F/MF^c^ and F/F^d^
				TEZ^a^(100 mg)				
				IVA^b^(300 mg)				
Keating	2018	Multicenter	4 weeks	VX-445(ELX) **(**200 mg)	Triple placebo or Placebo + TEZ(100 mg)+IVA(300 mg)	42	19	F/MF and F/F
				TEZ(100 mg)				
				TEZ(100 mg)				
Heijerman	2019	Multicenter	4 weeks	ELX^e^(200 mg)	TEZ(100 mg)+IVA(300 mg)	55	52	F/F
				TEZ(100 mg)				
				IVA(300 mg)				
Middleton	2019	Multicenter	24 weeks	ELX(200 mg)	Triple placebo	200	203	F/MF
				TEZ(100 mg)				
				IVA(300 mg)				
Barry	2021	Multicenter	8 weeks	ELX(200 mg)	TEZ(100 mg)+IVA(300 mg) or IVA(300 mg)	132	126	F-gating^f^/RF^g^
				TEZ(100 mg)				
				IVA(300 mg)				
Sutharsan	2021	Multicenter	24 weeks	ELX(200 mg)	TEZ(100 mg)+IVA(300 mg)	87	88	F/F
				TEZ(100 mg)				
				IVA(300 mg)				

a TEZ: tezacaftor.

b IVA: ivacaftor. .

c F/MF: p.Phe508del-minimal function.

d F/F: p.Phe508del-p.Phe508del.

e ELX: elexacaftor (VX-445)

f F-gating: p.Phe508del-gating.

g RF: p.Phe508del-residual function.

### Methodological Quality of the Included Studies (Risk of Bias)

The results of the assessment of the included RCTs are provided in Supplementary Table S3. All studies reported five aspects of bias (selection bias, performance bias, detection bias, attrition bias and reporting bias). All risks of bias in the included studies were low; therefore, the overall quality of included studies was considered high.

### Pooled Analysis of Primary Outcomes (ppFEV_1_, Sweat Chloride Concentration and CFQ-R Score) With Triple Placebo Comparator and F/MF Mutation

**FIGURE 2:**
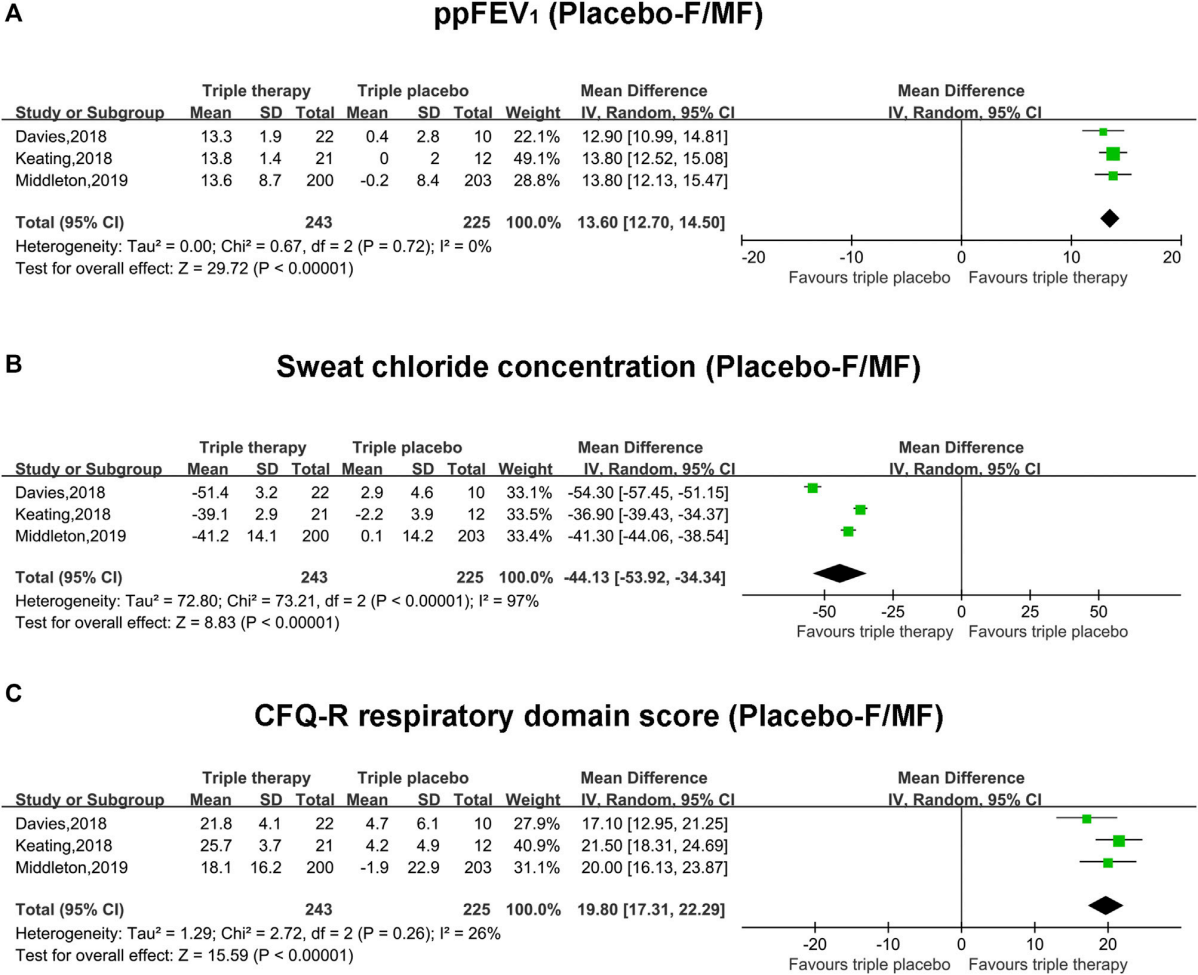
Forest plots of the included studies evaluating the efficacy of triple combination therapy vs. triple placebo with F/MF mutations. **(A)** ppFEV_1_. **(B)** Sweat chloride concentration. **(C)** CFQ-R respiratory domain score.

The pooled estimate of the absolute change in ppFEV_1_ in the triple combination therapy group was significantly higher than that of the triple placebo group (mean difference, MD, 13.6; 95% CI, 12.7–14.5), and the heterogeneity was significantly small (I^2^ = 0%) (Figure 2A). The pooled estimate of the absolute change in the sweat chloride concentration in the triple combination therapy group was clearly lower than that in the triple placebo group (MD, −44.13; 95% CI, −53.92 to −34.34); however, the heterogeneity was significantly high (I^2^ = 97%, *p* < 0.001) (Figure 2B). Moreover, the pooled outcome of CFQ-R was much higher in the triple combination therapy group than the triple placebo group (MD, 19.8; 95% CI, 17.31–22.29), with relatively unclear heterogeneity (I^2^ = 26%) (Figure 2C).

### Pooled Analysis of Primary Outcomes (ppFEV_1_, Sweat Chloride Concentration and CFQ-R Score) With Active Control Comparator and all Mutations and Subgroup Analysis of F/F Mutations

**FIGURE 3:**
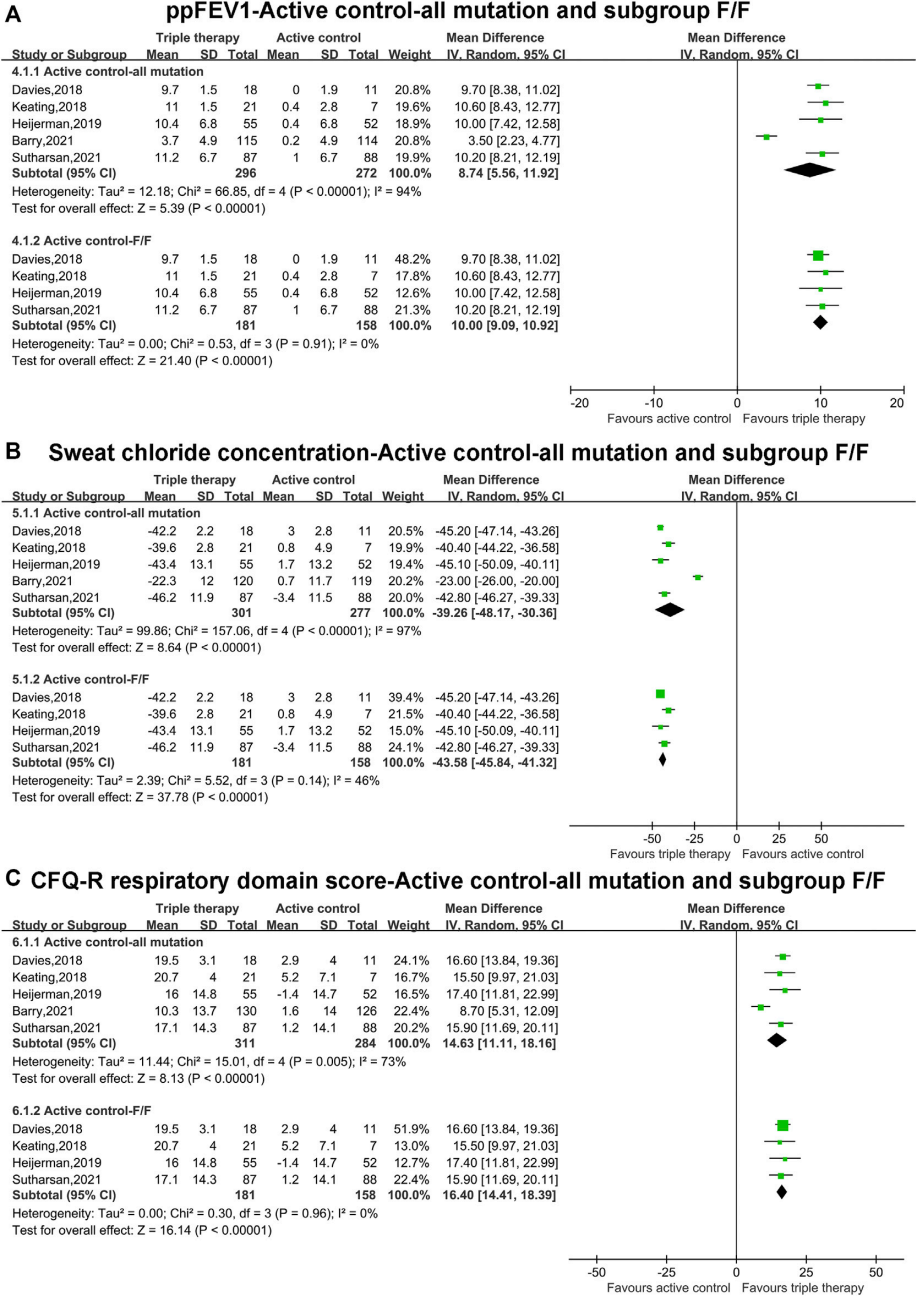
Forest plots of the included studies evaluating the efficacy of triple combination therapy vs. active control with all mutations and subgroup analysis of F/F mutations. **(A)** ppFEV_1_. **(B)** Sweat chloride concentration. **(C)** CFQ-R respiratory domain score.

The pooled estimate of the absolute change in ppFEV_1_ in the triple combination therapy group was significantly higher than that in the active group (MD, 8.74; 95% CI, 5.56–11.92), but the heterogeneity was significant (I^2^ = 94%, *p* < 0.001) (Figure 3A). After the data from Barry et al. (Barry et al., 2021), containing F-gating or RF mutations, were omitted, subgroup analysis was conducted in patients with F/F mutations. The pooled estimate of ppFEV_1_ in the triple combination therapy group was still higher than that in the active group (MD, 10.00; 95% CI, 9.09–10.92). Moreover, the heterogeneity became non-significant (I^2^ = 0%) (Figure 3A).

The pooled estimate of the absolute change in sweat chloride concentration in the triple combination therapy group was clearly lower than that in the active group (MD, −39.26; 95% CI, −48.17 to −30.36), with clear heterogeneity (I^2^ = 97%, *p* < 0.001) (Figure 3B). Subgroup analysis indicated that the pooled estimate of the sweat chloride concentration in the triple combination therapy group was lower than that in the active group in patients with F/F mutations (MD, −43.58; 95% CI, −45.84 to −41.32), and the heterogeneity was not clear (I^2^ = 46%) (Figure 3B).

The pooled outcome of the absolute change in CFQ-R in the triple combination therapy group was significantly higher than that in the active group (MD, 14.63; 95% CI, 11.11–18.16), and the heterogeneity was significant (I^2^ = 73%, *p* = 0.005) (Figure 3C). Subgroup analysis was conducted in patients with F/F mutations, and the pooled estimate of CFQ-R in the triple combination therapy group was still clearly higher than that in the active group (MD, 16.40; 95% CI, 14.41–18.39). In addition, the heterogeneity became non-significant (I^2^ = 0%) (Figure 3C).

### Adverse Events Between the Triple Combination Therapy Group and Placebo/Active Control Group

**FIGURE 4:**
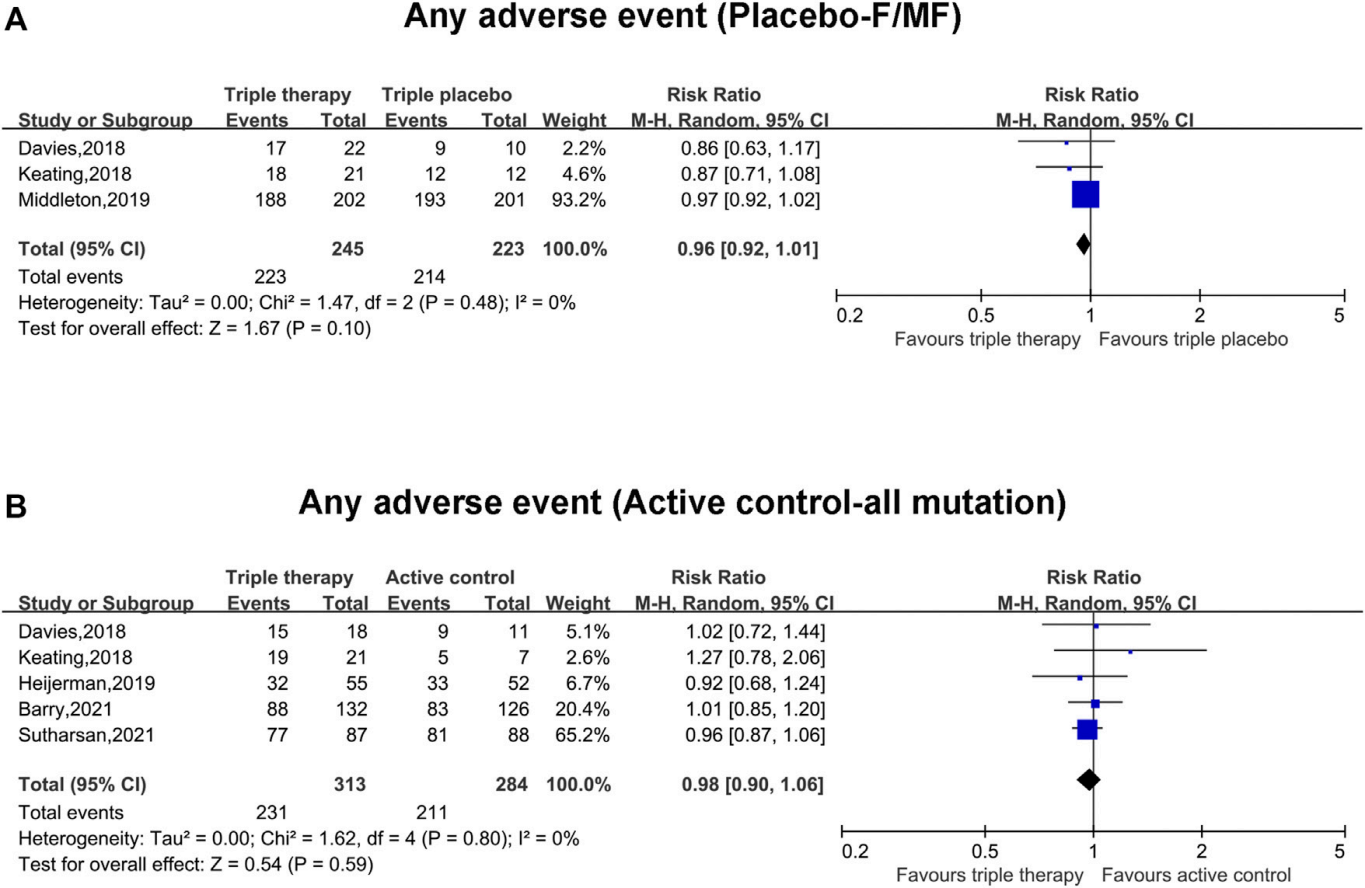
Forest plots of the included studies evaluating the safety according to any adverse events in triple combination therapy vs. triple placebo or active control. **(A)** Any adverse events (compared with triple placebo with F/MF). **(B)** Any adverse events (compared with active control with all mutations).

The pooled incidence of any adverse events in the triple combination therapy group was nearly the same as that in the placebo group (RR, 0.96; 95% CI, 0.92–1.01), with insignificant heterogeneity (I^2^ = 0%) (Figure 4A). Similarly, the pooled incidence of any adverse events in the triple combination therapy group was equivalent to that in the active group (RR, 0.98; 95% CI, 0.90–1.06), without clear heterogeneity (I^2^ = 0%) (Figure 4B).

Most of these adverse events were considered mild or moderate in the triple combination therapy group and placebo/active control group (Tables 2, 3). Furthermore, no clear differences were observed in adverse events leading to discontinuation of the trial regimen among the patients in the triple combination therapy group and placebo/active control group (Tables 2, 3). The most common adverse events (Tables 2, 3) were cough, infective pulmonary exacerbation of CF, headache, oropharyngeal pain, sputum increased and hemoptysis, which showed no clear difference between the triple combination therapy group and placebo/active control group (Tables 2, 3).

**TABLE 2:** Adverse event for placebo control with p.Phe508del-minimal function genotype.

Adverse event	Davies		Keating		Middleton	
	Number of patients (percent)					
	Triple therapy (*N* = 22)	Placebo (*N* = 10)	Triple therapy (*N* = 21)	Placebo (*N* = 12)	Triple therapy (*N* = 202)	Placebo (*N* = 201)
Any adverse event	17 (77)	9 (90)	18 (86)	12 (100)	188 (93.1)	193 (96.0)
Maximum severity of adverse event						
Mild	6 (35)	5 (56)	13 (72)	5 (42)	67 (33.2)	53 (26.4)
Moderate	10 (59)	4 (44)	5 (28)	6 (50)	102 (50.5)	125 (62.2)
Severe	1 (6)	0	0	1 (8)	19 (9.4)	14 (7.0)
Serious adverse event	1 (5)	3 (30)	0	2 (17)	28 (13.9)	42 (20.9)
Adverse event leading to discontinuation of the trial regimen	0	0	0	0	2 (1.0)	0
Most common adverse events						
Cough	4 (18)	1 (10)	7 (33)	1 (8)	34 (16.8)	77 (38.3)
Infective pulmonary exacerbation of cystic fibrosis	4 (18)	2 (20)	2 (10)	4 (33)	44 (21.8)	95 (47.3)
Headache	4 (18)	0	NA	NA	35 (17.3)	30 (14.9)
Oropharyngeal pain	4 (18)	0	NA	NA	20 (9.9)	25 (12.4)
Sputum increased	3 (14)	0	5 (24)	3 (25)	40 (19.8)	39 (19.4)
Hemoptysis	NA	NA	2 (10)	2 (17)	11 (5.4)	28 (13.9)

**TABLE 3:** Adverse event for active control with all mutation genotype.

Adverse event	Davies		Keating		Heijerman		Barry		Sutharsan	
	Number of patients (percent)									
	Triple therapy (*N* = 18)	Active control (*N* = 11)	Triple therapy (*N* = 21)	Active control (*N* = 7)	Triple therapy (*N* = 55)	Active control (*N* = 52)	Triple therapy (*N* = 132)	Active control (*N* = 126)	Triple therapy (*N* = 87)	Active control (*N* = 88)
Any adverse event	15 (83)	9 (82)	19 (90)	5 (71)	32 (58)	33 (63)	88 (66.7)	83 (65.9)	77 (89)	81 (92)
Maximum severity of adverse event										
Mild	7 (47)	2 (22)	10 (53)	2 (40)	23 (42)	21 (40)	58 (43.9)	50 (39.7)	48 (55)	46 (52)
Moderate	6 (40)	4 (44)	8 (42)	2 (40)	9 (16)	11 (21)	25 (18.9)	29 (23.0)	22 (25)	28 (32)
Severe	2 (13)	3 (33)	1 (5)	1 (20)	0	1 (2)	5 (3.8)	4 (3.2)	7 (8)	7 (8)
Serious adverse event	1 (6)	2 (18)	0	1 (14)	2 (4)	1 (2)	5 (3.8)	11 (8.7)	5 (6)	14 (16)
Adverse event leading to discontinuation of the trial regimen	0	0	1 (5)	1 (14)	0	0	1 (0.8)	2 (1.6)	2 (2)	1 (1)
Most common adverse events										
Cough	4 (22)	2 (18)	7 (33)	1 (14)	8 (15)	4 (8)	3 (2.3)	18 (14.3)	11 (13)	23 (26)
Infective pulmonary exacerbation of cystic fibrosis	5 (28)	3 (27)	5 (24)	1 (14)	1 (2)	6 (12)	3 (2.3)	13 (10.3)	10 (11)	36 (41)
Headache	3 (17)	0	NA	NA	3 (5)	4 (8)	11 (8.3)	19 (15.1)	25 (29)	18 (20)
Oropharyngeal pain	2 (11)	0	NA	NA	4 (7)	0	NA	NA	11 (13)	7 (8)
Sputum increased	3 (17)	1 (9)	8 (38)	0	NA	NA	NA	NA	10 (11)	16 (18)
Hemoptysis	NA	NA	3 (14)	0	2 (4)	5 (10)	NA	NA	NA	NA

## Discussion

Previous studies have revealed that monotherapy (ivacaftor), compared with placebo, improved the ppFEV_1_ in patients with Gly551Asp gating mutations (Ramsey et al., 2011). Subsequent studies have indicated that double combination therapy (corrector and potentiator, such as tezacaftor and ivacaftor), relative to placebo, improved ppFEV_1_, sweat chloride concentration and CFQ-R (Taylor-Cousar et al., 2017). However, not all double combination therapies have been found to effectively result in improvements in patients. Lumacaftor and ivacaftor slightly increased the ppFEV_1_ in patients with p.Phe508del homozygous mutation (Boyle et al., 2014; Wainwright et al., 2015), whereas no clinical benefits have been observed for patients with p.Phe508del heterozygous mutation (Boyle et al., 2014). A potential treatment rationale is that if the second mutation is responsive to ivacaftor alone, then double combination therapies may provide benefits (Meoli et al., 2021). Because the mechanism of the next-generation corrector differs from that of tezacaftor, the hypothesis that triple combination therapy would restore CFTR protein function has been suggested. In this meta-analysis, triple combination therapy was found to increase ppFEV_1_ by 13.6% relative to triple placebo in patients with F/MF mutations, with almost no heterogeneity. In the therapy group, as compared with the active control group, the ppFEV_1_ also markedly increased, by 8.74%; however, the heterogeneity was significant across studies. Clearly heterogeneous data came from Barry et al. (2021). After removal of the data from Barry et al., the heterogeneity of the pooled results clearly decreased. The CFTR mutations in Barry’s study were F-gating/RF, which are relatively less responsive to triple combination therapy. Sweat chloride concentration is the standard indicator of CFTR function (Middleton and Taylor-Cousar, 2021). The pooled sweat chloride concentration under triple combination therapy was much lower than that under triple placebo, thus indicating that triple therapy significantly restored the function of CFTR. Although the heterogeneity clearly came from Davies et al. (2018), the sweat chloride concentration in that study was much lower than those in the other two studies (Keating et al., 2018; Middleton et al., 2019). The next-generation corrector used in Davies et al. (2018) was VX659, and the effective data were extracted from the highest dose group (VX659 400 mg + TEZ + IVA), in contrast to Keating et al. (2018) and Middleton et al. (2019) (ELX 200 mg + TEZ + IVA). We attempted to use the data from a similar dose group (VX659 240 mg + TEZ + IVA) to decrease the heterogeneity; however, clear heterogeneity was still observed (I^2^ = 91%). Because the baseline demographic characteristics in the three patients were similar, the potential reason for the significant heterogeneity might have been that the structure and mechanism of VX659 differed from those of ELX. Fortunately, the presented effects VX659 were favorable for the patients. More studies are needed in the future to elucidate the specific mechanistic differences between VX659 and ELX. Similarly, triple combination therapy, in contrast to the active control, greatly decreased the pooled concentration of sweat chloride. The heterogeneity among studies might be explained by the data from Barry et al. (2021), which included F-gating/RF mutations. After exclusion of the heterogeneous data, the pooled results for sweat chloride concentration had only slight heterogeneity.

The CFQ-R respiratory domain score was used to evaluate the quality of life of patients with CF. The pooled results of the CFQ-R respiratory domain scores in the triple therapy combination were more satisfactory than those in the triple placebo group. Moreover, the consistency across studies was acceptable. The pooled estimate was also higher in the triple therapy combination group than the active control group; however, the data from Barry et al. (2021) clearly differed because of the inclusion of patients with F-gating/RF mutations. The heterogeneity decreased to insignificance in patients with only F/F mutations, and the pooled results were also elevated slightly.

Beyond the prominent benefits of the triple therapy combination, the safety was also favorable, as compared with that of placebo or active control, regardless of gene mutation type. The adverse events in the triple therapy combination group were nearly the same as those in the placebo or active control groups, with almost no heterogeneity. The specific adverse events (cough, infective pulmonary exacerbation, oropharyngeal pain, headache and increased sputum) were also similar between the triple therapy combination group and triple placebo or active control groups. Moreover, no dose-responsive relationship in adverse events was seen with the triple therapy combination (Davies et al., 2018; Keating et al., 2018). Overall, the safety of the triple therapy combination was similar to that in previous studies of CFTR modulators (Wainwright et al., 2015; Rowe et al., 2017; Taylor-Cousar et al., 2017). Hence, triple therapy combination appeared to achieve efficacy and safety simultaneously.

A recent systematic review about the efficacy and safety of CFTR modulators was conducted by Gramegna et al. (2020). The authors provided a comprehensive review of clinical results for monotherapy, dual combination and triple combination in CF patients with various genotyoe mutations. They concluded that CF patients with one gating mutation receiving IVA can benefit mostly in lung function, moreover, CF patients with homozygous or heterozygous p.Phe508del receiving ELX/TEZ/IVA can benefit in lung function, pulmonary exacerbation decrease and symptom improvement (Gramegna et al., 2020). Due to the multiple mixed comparisons in Gramegna’s research, they only made qualitative synthesis. By contrast, a quantitative synthesis (meta-analysis) is conducted in this research, which could manifest a pooled estimate for efficacy and safety of triple therapy combination compared with placebo and active-control group. Moreover, the result of NCT04058353 (which was ongoing when Gramegna’s article published) is included in our meta-analysis, confirming triple combination could offer additional benefit relative to previous CFTR modulators (Barry et al., 2021).

To our knowledge, this study is the first meta-analysis evaluating the efficacy and safety of the triple therapy combination in treating CF. The strengths of this meta-analysis were as follows. First, all included studies were multicenter RCTs, thus minimizing bias within the studies. Second, the comparison was conducted according to the type of control group (triple placebo or active control) and the type of mutation (F/MF or F/F); hence, the heterogeneity among the studies was as low as possible. Third, no clear adverse events were found in the triple therapy combination group, thus providing a basis for larger RCTs in the future.

Despite the advantages of triple combination therapy, some limitations of this study should also be considered: First, all patients included in the studies were 12 years or older, and data on the safety and efficacy of triple therapy combination in patients younger than 12 years were limited. However, a recent phase 3 open-label study has indicated that the treatment was safe and efficacious in children 6–11 years of age with at least one F508del-CFTR allele, thus supporting its use in this patient population (Zemanick et al., 2021). Furthermore, if the triple therapy combination does not have any significant safety issues in younger patients, the therapy is likely to be commenced in children after newborn screening, before the development of clinical disease (Middleton and Taylor-Cousar, 2021). Second, the mutation types differed in the included studies with an active control group, thus resulting in clear heterogeneity. However, the final effects were consistent across the included studies, and the difference was only in the extent of response to the triple therapy combination. In fact, researchers expect highly effective therapies to be available for all patients with CF, regardless of their variants, in the near future (Middleton and Taylor-Cousar, 2021). Third, the results from the included studies were mostly short-term; however, two included studies (Middleton et al., 2019; Sutharsan et al., 2021) used triple therapy for a relatively long period (24 weeks). Additional long-term results remain necessary to confirm the results.

In conclusion, the triple therapy combination had highly significant efficacy and safety in treating CF, as compared with placebo or active control, for patients with F/F, F/MF, F/RF or F-gating mutations. More well-designed RCTs are needed to support the efficacy and safety, and extend the indications for younger patients diagnosed with CF, to achieve radical treatment for CF before the development of the disease.

## Data Availability

The original contributions presented in the study are included in the article/Supplementary Material, further inquiries can be directed to the corresponding author.

## References

[B1] Barry P. J. Mall M. A. Álvarez A. Colombo C. de Winter-de Groot K. M. Fajac I. (2021). Triple Therapy for Cystic Fibrosis Phe508del-Gating and -Residual Function Genotypes. N. Engl. J. Med. 385 (9), 815–825. 10.1056/NEJMoa2100665 34437784 PMC8982185

[B2] Becq F. Mirval S. Carrez T. Lévêque M. Billet A. Coraux C. (2022). The rescue of F508del-CFTR by Elexacaftor/tezacaftor/ivacaftor (Trikafta) in Human Airway Epithelial Cells Is Underestimated Due to the Presence of Ivacaftor. Eur. Respir. J. 59 (2), 2100671. 10.1183/13993003.00671-2021 34266939

[B3] Boyle M. P. Bell S. C. Konstan M. W. McColley S. A. Rowe S. M. Rietschel E. (2014). A CFTR corrector (lumacaftor) and a CFTR potentiator (ivacaftor) for treatment of patients with cystic fibrosis who have a phe508del CFTR mutation: a phase 2 randomised controlled trial. Lancet Respir. Med. 2 (7), 527–538. 10.1016/S2213-2600(14)70132-8 24973281

[B4] Dalemans W. Barbry P. Champigny G. Jallat S. Dott K. Dreyer D. (1991). Altered Chloride Ion Channel Kinetics Associated with the delta F508 Cystic Fibrosis Mutation. Nature 354 (6354), 526–528. 10.1038/354526a0 1722027

[B5] Davies J. C. Moskowitz S. M. Brown C. Horsley A. Mall M. A. McKone E. F. (2018). VX-659-Tezacaftor-Ivacaftor in Patients with Cystic Fibrosis and One or Two Phe508del Alleles. N. Engl. J. Med. 379 (17), 1599–1611. 10.1056/NEJMoa1807119 30334693 PMC6277022

[B6] De Boeck K. Munck A. Walker S. Faro A. Hiatt P. Gilmartin G. (2014). Efficacy and Safety of Ivacaftor in Patients with Cystic Fibrosis and a Non-G551D Gating Mutation. J. Cyst Fibros 13 (6), 674–680. 10.1016/j.jcf.2014.09.005 25266159

[B7] Elborn J. S. (2016). Cystic Fibrosis. The Lancet 388 (10059), 2519–2531. 10.1016/s0140-6736(16)00576-6 27140670

[B8] Gramegna A. Contarini M. Aliberti S. Casciaro R. Blasi F. Castellani C. (2020). From Ivacaftor to Triple Combination: A Systematic Review of Efficacy and Safety of CFTR Modulators in People with Cystic Fibrosis. Int. J. Mol. Sci. 21 (16), 5882. 10.3390/ijms21165882 PMC7461566 32824306

[B9] Habib A. R. Kajbafzadeh M. Desai S. Yang C. L. Skolnik K. Quon B. S. (2019). A Systematic Review of the Clinical Efficacy and Safety of CFTR Modulators in Cystic Fibrosis. Sci. Rep. 9 (1), 7234. 10.1038/s41598-019-43652-2 31076617 PMC6510767

[B10] Heijerman H. G. M. McKone E. F. Downey D. G. Van Braeckel E. Rowe S. M. Tullis E. (2019). Efficacy and safety of the elexacaftor plus tezacaftor plus ivacaftor combination regimen in people with cystic fibrosis homozygous for the F508del mutation: a double-blind, randomised, phase 3 trial. Lancet 394 (10212), 1940–1948. 10.1016/S0140-6736(19)32597-8 31679946 PMC7571408

[B11] Higgins J. Green S. (2013). Cochrane Handbook for Systematic Reviews of Interventions, Version 5.1.0. London, United Kingdom: The Cochrane Collaboration.

[B12] Higgins J. P. Thompson S. G. Deeks J. J. Altman D. G. (2003). Measuring Inconsistency in Meta-Analyses. BMJ 327 (7414), 557–560. 10.1136/bmj.327.7414.557 12958120 PMC192859

[B13] Keating D. Marigowda G. Burr L. Daines C. Mall M. A. McKone E. F. (2018). VX-445-Tezacaftor-Ivacaftor in Patients with Cystic Fibrosis and One or Two Phe508del Alleles. N. Engl. J. Med. 379 (17), 1612–1620. 10.1056/NEJMoa1807120 30334692 PMC6289290

[B14] Meoli A. Fainardi V. Deolmi M. Chiopris G. Marinelli F. Caminiti C. (2021). State of the Art on Approved Cystic Fibrosis Transmembrane Conductance Regulator (CFTR) Modulators and Triple-Combination Therapy. Pharmaceuticals (Basel) 14 (9), 1–21. 10.3390/ph14090928 PMC8471029 34577628

[B15] Middleton P. G. Mall M. A. Dřevínek P. Lands L. C. McKone E. F. Polineni D. (2019). Elexacaftor-Tezacaftor-Ivacaftor for Cystic Fibrosis with a Single Phe508del Allele. N. Engl. J. Med. 381 (19), 1809–1819. 10.1056/NEJMoa1908639 31697873 PMC7282384

[B16] Middleton P. G. Taylor-Cousar J. L. (2021). Development of Elexacaftor - Tezacaftor - Ivacaftor: Highly Effective CFTR Modulation for the Majority of People with Cystic Fibrosis. Expert Rev. Respir. Med. 15 (6), 723–735. 10.1080/17476348.2021.1855980 33249928

[B17] Ramsey B. W. Davies J. McElvaney N. G. Tullis E. Bell S. C. Dřevínek P. (2011). A CFTR Potentiator in Patients with Cystic Fibrosis and the G551D Mutation. N. Engl. J. Med. 365 (18), 1663–1672. 10.1056/NEJMoa1105185 22047557 PMC3230303

[B18] Rowe S. M. Daines C. Ringshausen F. C. Kerem E. Wilson J. Tullis E. (2017). Tezacaftor-Ivacaftor in Residual-Function Heterozygotes with Cystic Fibrosis. N. Engl. J. Med. 377 (21), 2024–2035. 10.1056/NEJMoa1709847 29099333 PMC6472479

[B19] Sutharsan S. McKone E. F. Downey D. G. Duckers J. MacGregor G. Tullis E. (2021). Efficacy and Safety of Elexacaftor Plus Tezacaftor Plus Ivacaftor versus Tezacaftor Plus Ivacaftor in People with Cystic Fibrosis Homozygous for F508del-CFTR: a 24-week, Multicentre, Randomised, Double-Blind, Active-Controlled, Phase 3b Trial. Lancet Respir. Med. 10.1016/s2213-2600(21)00454-9 34942085

[B20] Taylor-Cousar J. L. Munck A. McKone E. F. van der Ent C. K. Moeller A. Simard C. (2017). Tezacaftor-Ivacaftor in Patients with Cystic Fibrosis Homozygous for Phe508del. N. Engl. J. Med. 377 (21), 2013–2023. 10.1056/NEJMoa1709846 29099344

[B21] Veit G. Roldan A. Hancock M. A. Da Fonte D. F. Xu H. Hussein M. (2020). Allosteric folding correction of F508del and rare CFTR mutants by elexacaftor-tezacaftor-ivacaftor (Trikafta) combination. JCI insight 5 (18), 1–21. 10.1172/jci.insight.139983 PMC7526550 32853178

[B22] Vrabel M. (2009). Preferred Reporting Items for Systematic Reviews and Meta-Analyses: The PRISMA Statement. Revista Espaola De Nutrición Humana Y Dietética 18 (3), e123. 10.14306/renhyd.18.3.114 PMC3090117 21603045

[B23] Wainwright C. E. Elborn J. S. Ramsey B. W. Marigowda G. Huang X. Cipolli M. (2015). Lumacaftor-Ivacaftor in Patients with Cystic Fibrosis Homozygous for Phe508del CFTR. N. Engl. J. Med. 373 (3), 220–231. 10.1056/NEJMoa1409547 25981758 PMC4764353

[B24] Zemanick E. T. Taylor-Cousar J. L. Davies J. Gibson R. L. Mall M. A. McKone E. F. (2021). A Phase 3 Open-Label Study of Elexacaftor/Tezacaftor/Ivacaftor in Children 6 through 11 Years of Age with Cystic Fibrosis and at Least One F508del Allele. Am. J. Respir. Crit. Care Med. 203 (12), 1522–1532. 10.1164/rccm.202102-0509OC 33734030 PMC8483230

