# Supplementary material for: Efficacy and Safety of Triple Combination Cystic Fibrosis Transmembrane Conductance Regulator Modulators in Patients With Cystic Fibrosis: A Meta-Analysis of Randomized Controlled Trials
Source: Front Pharmacol. 2022 Mar 14;13:863280. doi: 10.3389/fphar.2022.863280 (PMC8964016; doi:10.3389/fphar.2022.863280)
Supplement: Supplementary file 3 [file Table3.DOCX]

**Supplementary Table 3 Risk of bias assessment of RCTs using the Cochrane Collaboration tool**

| **Study** | **Random sequence generation** | **Allocation concealment** | **Performance bias** | **Detection bias** | **Attrition bias** | **Reporting bias** |
| --- | --- | --- | --- | --- | --- | --- |
| Davies | Low | Low | Low | Low | Low | Low |
| Keating | Low | Low | Low | Low | Low | Low |
| Heijerman | Low | Low | Low | Low | Low | Low |
| Middleton | Low | Low | Low | Low | Low | Low |
| Barry | Low | Low | Low | Low | Low | Low |

RCT, Randomized controlled trial
